# Measuring the Carboxypeptidase and γ-Glutamyltranspeptidase Activities of Lager and Ale Yeasts to Assess Their Impact on the Release of Odorant Polyfunctional Thiols Through Fermentation

**DOI:** 10.3390/molecules30122491

**Published:** 2025-06-06

**Authors:** Coraline Calicis, Romain Christiaens, Natacha Loquet, Margaux Simon, Sonia Collin

**Affiliations:** Unité de Brasserie et des Industries Alimentaires, Louvain Institute of Biomolecular Science and Technology (LIBST), Faculté des Bioingénieurs, Université Catholique de Louvain, Croix du Sud, 2 box L7.05.07, 1348 Louvain-la-Neuve, Belgium; coraline.calicis@uclouvain.be (C.C.); romain.christiaens@uclouvain.be (R.C.); natacha.loquet@student.uclouvain.be (N.L.); margaux.simon@uclouvain.be (M.S.)

**Keywords:** beer, flavor, odorant thiols, hop, glutathionylated precursors

## Abstract

Two enzymatic assays, based on release of *p*-nitroaniline and its spectrophotometric detection at 405 nm, were used to screen lager and ale brewing yeasts for carboxypeptidase and γ-glutamyltranspeptidase activity. Both activities were found in all the investigated yeasts and did not significantly distinguish *Saccharomyces cerevisiae* from *S. pastorianus* species. Large between-strain differences were measured for both carboxypeptidase (from 1.61 A/h for BRAS-45 to 41.71 A/h for E-30) and γ-glutamyltranspeptidase (from 1.26 A/h for US-05 to 48.72 A/h for S-33). No correlation was found between either enzymatic activity and the previously published ability of *Saccharomyces* yeasts to degrade glutathionyl or γ-GluCys- precursors to free polyfunctional thiols. Only for fermentation at lower temperatures does carboxypeptidase activity seem relevant for identifying the most interesting candidates. Measuring transport efficiency and β-lyase activities individually on the three possible intermediates emerges here as more promising for future flavor potential screening.

## 1. Introduction

In today’s context, the depletion and high cost of raw materials compel industries to optimize resource use. In brewing, one key strategy is to improve bioconversion of the sulfur-containing aroma precursors found in hops and malt to take full advantage of these natural flavor resources [1,2]. Indeed, because of alkenal detoxification by glutathione (GSH), glutathionylated (G-) precursors and their derived cysteinylated (Cys-) adducts are found in much higher quantities than free thiols. For example, G-3SHol, the glutathionylated precursor of 3-sulfanylhexanol (3SHol, rhubarb and passionfruit odor), can reach up to 100 mg/kg in Amarillo hops [3] and up to 300 µg/kg in pale malts [1,3]. Although precursors from malt seem to be lost in the spent grain, late and dry hopping have emerged as techniques contributing to substantial amounts of pleasant polyfunctional thiols (PFTs) in beer [4]. Before cleavage of the β C-S bond, G- and Cys- precursors are not detectable by the human organoleptic system [5]. Among the different matrices containing bound and free PFTs, hop remains the most challenging, with a diversity of up to 41 different molecules [4,6]. PFT release from precursors is catalyzed by enzymatic activities identified in mammalian secretions (e.g., cat urine, human saliva, and sweat), plants, microflora, and yeast strains used for fermenting beer or wine [7,8,9,10].

During yeast fermentation, glutathione conjugates are taken up by the cell through Opt1p, a high-affinity plasma membrane GSH transporter. To neutralize these potentially toxic compounds, they are subsequently transported into the vacuole via specialized transporters such as Ycf1p and Bpt1p [11,12]. Inside the vacuole, dipeptides are released through hydrolysis by peptidases, such as γ-glutamyltranspeptidase (γ-GT) encoded by *ECM38*, or by a carboxypeptidase (CPY) encoded by *PRC1* [12,13]. In yeast, the pathway initiated by the CPY is preferred [14]. This enzyme cleaves the glycine residue from the dipeptide, forming γ-GluCys-. Subsequently, γ-GT removes the γ-glutamate residue, resulting in the formation of the Cys- precursor. The alternative pathway, initiated by γ-GT activity leading to the release of CysGly-, should also be considered. However, it is important to note that γ-GT exhibits optimal activity at a pH of 8 to 8.5, while the vacuolar pH is around 5.2, which may limit its efficiency in this compartment—especially considering that CPY is active under acidic conditions [15,16]. In wine and beer, it is now well recognized that yeast β-lyase can cleave the sulfur–carbon bond of Cys- conjugates to release free PFTs [9]. Transporters such as Gap1p and Ptr2p also regulate the transport of dipeptides and Cys- adducts into and from the cell, optimizing the release of volatile thiols [13].

In the brewing field, among various tested ale yeasts (*S. cerevisiae*), SafAle^TM^ K-97 was recently shown to exhibit the highest β-lyase activity on Cys- conjugates, reaching up to 0.54% release from Cys-3SHol. On the other hand, very poor release from the G- precursor was observed with this strain [17]. Yeast SafAle^TM^ S-33, the second-best ale in terms of release efficiency, showed superior acetyltransferase activity, esterifying up to 80% of the alcohols released, especially from Cys-3-sulfanylpentanol (Cys-3SPol) and Cys-3-sulfanyl-4-methylpentanol (Cys-3S4MPol) [17]. Among ale yeasts, a maltose-negative *S. cerevisiae* var. *chevalieri* strain (SafBrew^TM^ LA-01) emerged as the only one able to better release free PFT from G- than from Cys-3SHol. This suggests direct β-lyase activity on the tripeptide [18]. On the other hand, many *S. pastorianus* strains (used for lager beers) appear to cleave the tripeptide precursor more efficiently than its cysteinylated counterpart [19]. Very recently, some lager strains were even found to cleave the dipeptide intermediate γ-GluCys-3SHol (rates achieved: 0.45% with BRAS-45 and 0.28% with E-30) more efficiently than G-3SHol (rate achieved: 0.21% with BRAS-45) [8,20].

The aim of the present study was to develop quick enzymatic assays for profiling the γ-GT and carboxypeptidase activities of different yeast strains.

## 2. Results and Discussion

### 2.1. New Enzymatic Assay for Comparing γ-GT Activity in Various Lager and Ale Brewing Yeasts

A new protocol was established to compare γ-GT activity in various lager and ale brewing yeasts. The first step was to extract the γ-GT enzyme, located on the inner membrane of the vacuole. YPS medium containing the yeast cells was mixed with glass beads to disrupt the cells and release their content. This was followed by successive centrifugations to obtain a fraction containing all proteins and organelles.

**Figure 1:**
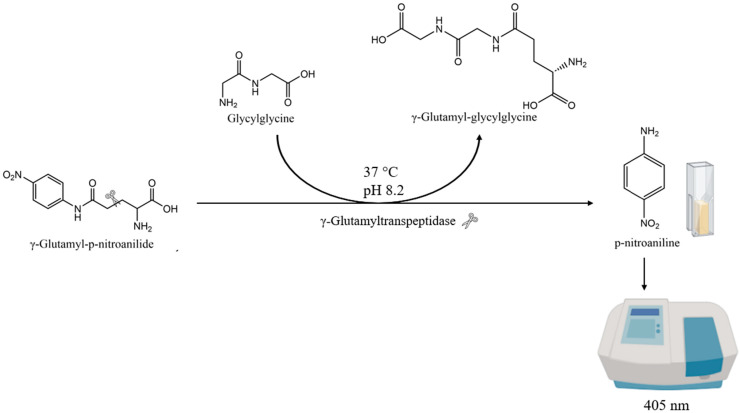
Scheme of the γ-GT activity enzymatic assay.

In a second step, the enzymatic activity was measured at 405 nm on γ-glutamyl-*p*-nitroanilide as the substrate [21] (Figure 1). Glycylglycine was used as the γ-glutamyl moiety acceptor in this enzymatic assay conducted at 37 °C and pH 8.2. Specific γ-GT activity was not investigated here, because of the predominance of proteins other than γ-GT in the enzymatic extracts. Data are given in A/h (slope of the absorbance increase per hour) for each yeast extracted in the same way from 10^8^ cells/mL in 200 mL YPS medium. As a control, glutathione, the primary natural substrate of γ-GT, was added as a competitor, to confirm that the observed activity was specifically due to γ-GT, as previously proposed by Penninckx et al. [22]. This approach not only confirmed the specificity of the observed activity but also showed that γ-GT activity on PFT precursors could be reduced by approximately half in the presence of 31 mM glutathione in beer (Figure S1).

**Figure 2:**
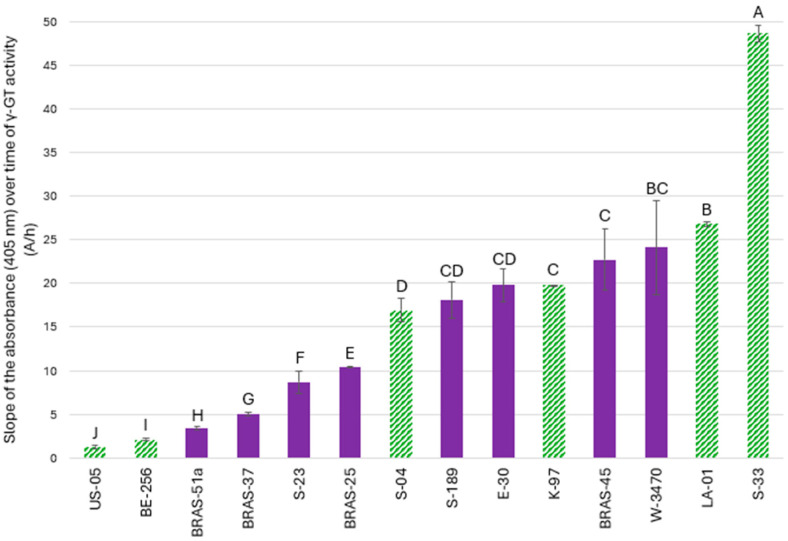
Slopes of the absorbance (405 nm) over time (A/h) for γ-GT activity in all the lager (full purple) and ale (dashed green) brewing yeasts tested. Values with different letters are significantly different (*p* < 0.05) according to the Student–Newman–Keuls test.

As depicted in Figure 2, γ-GT activity was measured in eight lager yeasts, five ale yeasts, and one maltose-negative strain (*S. cerevisiae* SafAle™ LA-01). The ale yeast SafAle™ S-33 exhibited the highest γ-GT activity (48.72 A/h), followed by the maltose-negative yeast SafAle™ LA-01 (26.82 A/h). Yet, γ-GT activity did not significantly distinguish *S. cerevisiae* from *S. pastorianus* strains, even if the ale yeast strain yeast SafAle™ S-33 stood out from the others. The most active lager strain (SafLager™ W-3470) showed a considerably lower activity, with only 24.11 A/h.

### 2.2. Enzymatic Assay for Comparing the Carboxypeptidase Activities of Various Lager and Ale Brewing Yeasts

With carboxypeptidase being an intravacuolar enzyme, this assay required using a reagent to facilitate its release. Proteins were solubilized in cell lysis buffer (CelLytic^TM^ Y Cell) as in Easwaran et al. [23]. The resulting solution was centrifuged and the supernatant collected.

**Figure 3:**
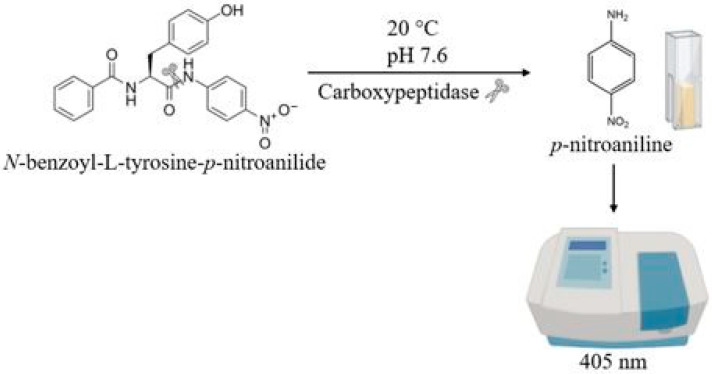
Scheme of the carboxypeptidase enzymatic assay.

For the enzymatic assay, *N*-benzoyl-L-tyrosine-*p*-nitroanilide (NBT*p*NA) was used as a substrate, as in Ramirez-Zavala et al. [24] (Figure 3). Carboxypeptidase activity was assessed by measuring the increase in *p*-nitroaniline absorbance at 405 nm. The tests were carried out at 20 °C and pH 7.6. As mentioned above for γ-GT, because of the predominance of proteins other than carboxypeptidase in the enzymatic extract, we preferred not to translate our raw data (obtained for 10^8^ cells/mL in 200 mL YPS medium) to specific activity. As a control, a commercial carboxypeptidase (Sigma-Aldrich: Saint Louis, MO, USA) was also tested.

**Figure 4:**
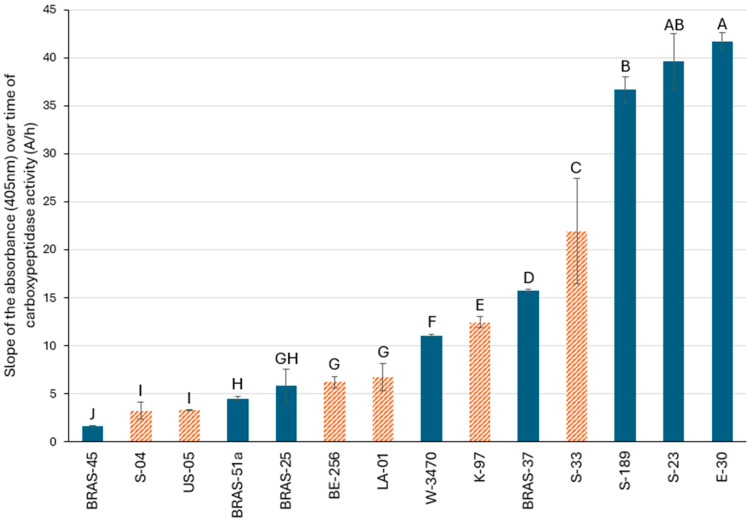
Slope of the absorbance (405 nm) over time (A/h) for carboxypeptidase activity in all the lager (full blue) and ale (dashed orange) yeasts tested. Values with different letters are significantly different (*p* < 0.05) according to the Student–Newman–Keuls test.

As depicted in Figure 4, carboxypeptidase activity was measured in eight lager yeasts, five ale yeasts, and one maltose-negative strain (*S. cerevisiae* SafAle™ LA-01). The lager yeast SafLager™ E-30 exhibited the highest carboxypeptidase activity (41.71 A/h), followed by SafLager™ S-23 (39.62 A/h). As mentioned above for γ-GT, no significant difference in carboxypeptidase activity was observed between lager and ale brewing yeasts. However, the three lager yeasts SafLager™ E30, SafLager™ S23, and SafLager™ S-189 stood out from the other yeasts by exhibiting higher activity (above 35 A/h), whereas the first ale strain (SafAle™ S-33) showed an activity of only 21.94 A/h.

### 2.3. Investigation of Whether, in Lager Yeasts, There Is a Relationship Between γ-GT or Carboxypeptidase Activity and the Ability to Release Free PFTs from S Conjugates, Especially γ-GluCys-Precursors

We then investigated, for a few selected strains (BRAS-45, E-30, and BRAS-51a), whether the γ-GT and carboxypeptidase activities measured here might explain why some of them are so efficient in terms of PFT release from the dipeptide precursor γ-GluCys-3SHol (data previously published [20]).

**Figure 5:**
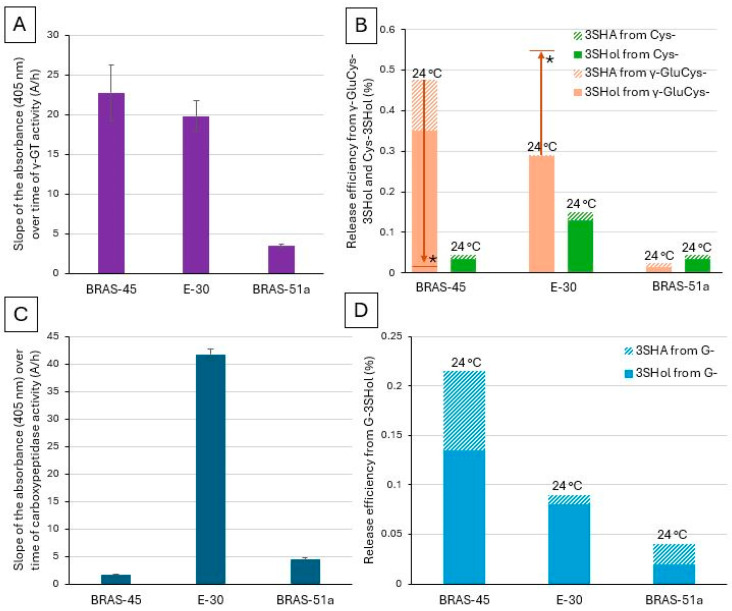
(**A**) γ-GT activity—slope of the absorbance (405 nm) over time (A/h). (**B**) Efficiency (%) of free 3SHol (full) and 3SHA (dashed) release from γ-GluCys- (orange) and Cys- (green) precursors at 24 °C (or 12 °C *) (data previously published [19,20]). (**C**) Carboxypeptidase activity—slope of the absorbance (405 nm) over time (A/h). (**D**) Efficiency (%) of free 3SHol (full) and 3SHA (dashed) release at 24 °C from G- precursors (blue) (data previously published [19]). Each value is the mean of duplicate assays.

Among lager yeasts, BRAS-45 and E-30 demonstrated relatively high γ-GT activity, reaching 22.73 A/h and 19.79 A/h, respectively (Figure 5A). At first glance, this might explain why they release free PFTs so efficiently from γ-GluCys-3SHol (0.48% and 0.29% for BRAS-45 and E-30, respectively). Indeed, high γ-GT activity enables the efficient conversion of γ-GluCys-3SHol into Cys-3SHol, which could be subsequently degraded by a β-lyase into free PFTs. However, as illustrated in Figure 5B, both strains release free PFTs much more efficiently from γ-GluCys-3SHol than from Cys-3SHol (only 0.045% and 0.15%, respectively). Thus, the role of γ-GT in releasing PFTs from G- and γ-GluCys-precursors appears very minor in lager yeasts, given their low β-lyase activity on Cys- conjugates. This suggests that the pathway involving a β-lyase acting directly on γ-GluCys-3SHol is probably more efficient than the coupled γ-GT/β-lyase activities required with a β-lyase active only on the Cys- intermediate (as usually implied in the literature [18]). Yet another explanation could be a preference for lager yeasts to take tri- and dipeptides from the wort instead of the Cys- precursor.

In the same way, no correlation was observed between the carboxypeptidase activity measured here and the efficiency of PFT release from G-3SHol (Figure 5C,D). BRAS-45 exhibited very low carboxypeptidase activity despite its higher efficiency of free PFT release from G-3SHol, with the opposite being true for E-30. This indicates that the main enzymatic activity responsible for release of free PFTs is attributable to β-lyases acting mainly, in the case of lager yeasts, on G- and γ-GluCys-3SHol.

Very intriguing, however, is the very high carboxypeptidase activity measured for E-30, the only investigated lager strain, having shown much higher release from γ-GluCys-3SHol at 12 °C than at 24 °C (S-23 and S-189, unfortunately, were not screened in this study). We suspected that only the β-lyase activity on the dipeptide precursor remained efficient at 12 °C [20]. Hence, at 12 °C the carboxypeptidase of E-30 might allow for producing significant amounts of the dipeptide γ-GluCys- intermediate from G-precursors (the major PFT pool in hops), with subsequent thiol release.

Our results suggest that the next step should be to develop a quick assay for screening lager yeasts for β-lyase activity (most probably their limiting enzymatic step for PFT release) individually on G-, γ-GluCys- and Cys- precursors. The data obtained will have to be discussed in relation to the relative amount of each precursor in authentic worts.

### 2.4. Investigation of Whether There Is a Relationship Between the γ-GT or Carboxypeptidase Activity of Ale Yeasts and Their Ability to Release Free PFTs from G-Conjugates

**Figure 6:**
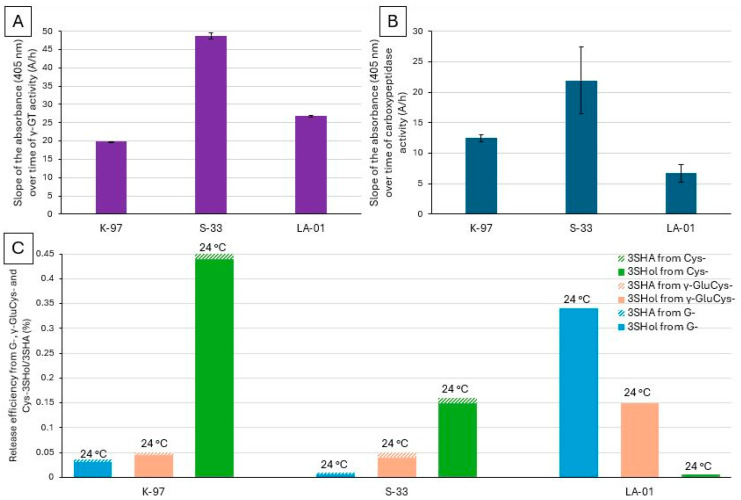
(**A**) γ-GT and (**B**) carboxypeptidase activity—slope of the absorbance (405 nm) over time (A/h). (**C**) Efficiency (%), at 24 °C, of free 3SHol (full) and 3SHA (dashed) release from G- (light blue), γ-GluCys- (orange), and Cys-precursors (green) (data previously published [15,16,18]). Results are means of duplicate assays.

Among ale yeasts, K-97 and S-33 showed γ-GT activities (19.83 A/h and 48.72 A/h, respectively) similar (or higher) to those of the best lager yeasts (Figure 2 and Figure 6A). They thus appear sufficiently equipped to degrade G-3SHol to CysGly-3SHol or γ-GluCys-3SHol to Cys-3SHol. As depicted in Figure 4 and Figure 6B, both also showed substantial carboxypeptidase activity (up to 12.45 A/h for K-97 and 21.94 A/h for S-33). Moreover, of all the yeasts previously screened, K-97 had the highest β-lyase activity, reaching up to 0.45% efficiency of release from Cys- precursors (Figure 6C). Again, active transport of bigger precursors into the cell could be another limiting factor.

Neither of these strains, however, showed efficient release of free PFT from G-3SHol (0.035% release efficiency for K-97 and 0.01% for S-33) or γ-GluCys-3SHol (0.05% for both K-97 and S-33) (Figure 6C). This suggests that their β-lyase requires a smaller substrate than tri- or dipeptides, such as Cys-3SHol. From the tripeptide, therefore, these ale yeasts have to follow the full degradation pathway, using both their carboxypeptidase and γ-GT activities to produce Cys- precursors from which their β-lyase releases free PFTs. 

Among the ale yeasts tested, the maltose–maltotriose-negative yeast strain *S. cerevisiae* var. *chevalieri* (LA-01) exhibits moderate carboxypeptidase activity (6.73 A/h) and high γ-GT activity (26.82 A/h) (Figure 6A,B). It appears to release PFTs much more efficiently from G-precursors (0.34%) than from γ-GluCys- and Cys- precursors (0.15% and 0.006%, respectively) (Figure 6C). This suggests, as for lager strains, direct β-lyase activity on the tripeptide. LA-01 is therefore of particular interest for producing low/non-alcoholic beer with a great aromatic profile [20].

**Figure 7:**
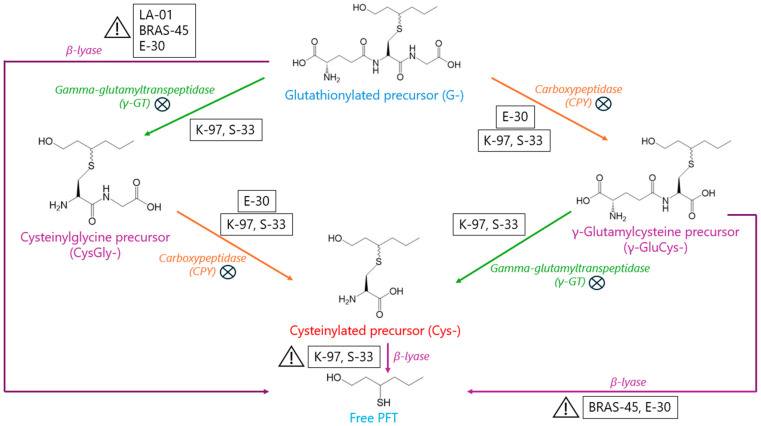
Suspected key pathways (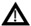) and non-limiting factors (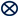) in the release, by brewing yeasts, of odorant PFTs from G, γ-GluCys-, and Cys- precursors.

In summary, Figure 7 shows the suspected enzymatic pathways used by the most interesting lager, ale, and maltose-negative strains investigated here. UPLC-MS/MS analyses of the different intermediates occurring in a fermenting wort should make it possible to refine these hypotheses.

## 3. Materials and Methods

### 3.1. Chemicals

Tris(hydroxymethyl)aminomethane (Tris-HCl), barium hydroxide octahydrate, sodium deoxycholate, *N*-benzoyl-L-tyrosine-*p*-nitroaniline (NBT*p*NA), CelLytic™ Y Cell, imidazole, magnesium chloride, d-sorbitol, dithiothreitol (DTT), γ-glutamyl-*p*-nitroanilide, dimethyl sulfoxide (DMSO), and glycylglycine were obtained from Sigma-Aldrich (Bornem, Belgium). Zinc sulfate was purchased from UCB (Leuven, Belgium). Dimethylformamide was acquired from Janssen Chimica (Beerse, Belgium). Phenylmethylsulphonyl fluoride (PMSF) was from Merck (Darmstadt, Germany).

### 3.2. Yeasts

Four lager yeasts (BRAS-25, BRAS-37, BRAS-45, BRAS-51a) from the INBr UCLouvain collection (Louvain-la-Neuve, Belgium), propagated in liquid yeast extract/peptone/sucrose (YPS) medium at 28 °C, and seven active dry yeasts (SafAle^TM^ S-33, SafAle^TM^ K-97, SafAle^TM^ US-05, SafAle^TM^ BE-256, SafAle^TM^ S-04, SafLager^TM^ S-23, SafLager^TM^ W34-70, SafLager^TM^ S-189, SafLager^TM^ E-30, and SafBrew^TM^ LA-01) from Fermentis Lesaffre (Marcq-en-Baroeul, France) were used in the fermentation trials. YPS aqueous medium was composed of MgCl_2_ (0.58 g/L), NaCl (0.5 g/L), (NH_4_)_2_SO_4_ (1 g/L), CaCl_2_·2H_2_O (0.1 g/L), KH_2_PO_4_ (1875 g/L), maltose syrup (0.017 g/L), sucrose (108.33 g/L), yeast extract (18.33 g/L), tryptone (2 g/L), Tween 80 (0.1 g/L), and FeCl (1 g/L).

### 3.3. γ-GT Activity Measurement

#### 3.3.1. γ-GT Extraction

On the basis of previous work performed by Delhez et al., 200 mL of YPS aqueous medium containing 10^8^ cells/mL was centrifuged at 6000 RPM (1610× *g*, r = 4 cm; Beckman Coulter AVANTI J-E) and 4 °C for 10 min [25]. The resulting pellet was washed with Milli-Q water and then centrifuged again under the same conditions (6000 RPM at 4 °C for 10 min). For each gram of the obtained pellet, we added 1.5 mL of pH 7 grinding medium containing imidazole (50 mM), sorbitol (250 mM), and MgCl_2_ (1 mM), along with PMSF (1 mM), DTT (10 mM), and two mixes of protease inhibitors (0.0005 mM of each; dilution 1:2000). One mix consisted of leupeptin, aprotinin, and antipain at 4 mg/mL in water, while the second contained chymostatin and pepstatin (4 mg/mL prepared in DMSO). A total of 3.5 mL glass beads (425 µm to 600 µm) was added and the solution was processed three times in a Precellys Evolution homogenizer (Bertin Technologies: Montigny-le-Bretonneux, France) (8500 RPM for 40 s), with a 1 min incubation on ice between successive cycles. After centrifugation at 4000 RPM for 5 min at 4 °C (716× *g*, r = 4 cm; Eppendorf centrifuge 5430), the recovered supernatant was subjected to another 5 min centrifugation at 5000 RPM and 4 °C before being frozen in liquid nitrogen and stored at −80 °C.

#### 3.3.2. γ-GT Enzymatic Assay

We mixed 650 µL Milli-Q water, 100 µL Tris-HCl buffer (1 M at pH 8.2 in Milli-Q water), 100 µL γ-glutamyl-*p*-nitroanilide (62.5 mM in DMSO), and 100 µL glycylglycine (0.5 M in Milli-Q water) before adding 50 µL enzymatic extract. Absorbance at 405 nm was measured for one hour at 37 °C (Genesys 10S UV-Vis Thermo Fisher Scientific: Madison, WI, USA).

For tests with glutathione, 550 µL Milli-Q water, 100 µL Tris-HCl (1 M at pH 8.2 in Milli-Q water), 100 µL γ-glutamyl-*p*-nitroanilide (62.5 mM in DMSO), 100 µL of GSH solution (62.5 mM in Milli-Q water), and 100 µL of glycylglycine (0.5 M in Milli-Q water) were mixed before adding 50 µL enzymatic extract. Absorbance at 405 nm was measured for one hour at 37 °C (Genesys 10S UV-Vis Thermo Scientific).

### 3.4. Carboxypeptidase Activity Measurement

#### 3.4.1. Carboxypeptidase Enzymatic Extraction

On the basis of previous work performed by Easwaran et al. [23], yeast cells from aqueous YPS medium containing 10^8^ cells/mL were collected after 10 min of centrifugation at 5000 RPM (1118× *g*, r = 4 cm; Sorvall RC-5B Refrigerated Superspeed Centrifuge). Cell lysis was carried out by treating the yeast cells with lysis buffer (CelLytic™ Y Cell) at 5 mL per gram of cells. The solution was incubated for 30 min on a rotating plate and then centrifuged at 5000 RPM for 10 min. The supernatant was stored at −80 °C for use in enzymatic assays.

#### 3.4.2. Carboxypeptidase Enzymatic Assay

The enzymatic activity of carboxypeptidase was quantified spectrophotometrically by measuring the release, catalyzed by this enzyme, of *p*-nitroanilide from the NBT*p*NA substrate. A solution containing 250 µL Tris-HCl (200 mM at pH 7.6 in Milli-Q water), 250 µL of 2% sodium deoxycholate, 10 µL NBT*p*NA (1 mM dissolved in dimethylformamide), and 500 µL enzymatic extract was incubated at 37 °C for 6 h. Absorbance measurements were taken every 30 min. Before each absorbance measurement, enzymatic activity was halted by adding stop solution consisting of 500 µL of 5% ZnSO_4_ and 100 µL of 7.5% Ba(OH)_2_. The mixture was then centrifuged for 10 min at 13000 RPM (7558× *g*, r = 4 cm; Sigma 1–14 K) and the absorbance of released *p*-nitroaniline was measured at 405 nm (VWR UV-3100PC).

### 3.5. Statistical Analysis

All analytical measurements were carried out in duplicate. Multiple comparisons of the means were performed with Student–Newman–Keuls tests (JMP Program 17). Values sharing no common letter are significantly different (*p* < 0.05).

## 4. Conclusions

The present study aimed at developing protocols to quickly assess the γ-GT and carboxypeptidase activities of brewing yeasts. For γ-GT, an effective method has been established, with direct protein extraction by glass bead milling at pH 8.2 in the presence of protease inhibitors. For carboxypeptidase protein extraction, the CelLytic^TM^ Y Cell was used. In both cases, enzymatic activity was assessed spectrophotometrically at 405 nm (absorbance wavelength of *p*-nitroaniline).

In lager yeasts, neither the γ-GT nor the carboxypeptidase activity emerged here as the limiting factor for free PFT release from S conjugates. Further research on the transport proteins involved in the import and export of precursors (G-, γ-Glu-Cys, and Cys-) across the cell membrane is needed to determine whether this step represents a limiting factor in the release of PFTs into the medium. Additionally, investigating the activity of β-lyase on these three precursors, along with UPLC-MS/MS analyses of the various intermediates present in fermenting wort, should help refine our hypotheses.

## Data Availability

Data are contained within this article or supplementary materials.

## Supplementary Materials

The following supporting information can be downloaded at https://www.mdpi.com/article/10.3390/molecules30122491/s1, Figure S1: Enzymatic assays of ƴ-GT activity of K-97 and E-30 in the presence of spiked glutathione (6.25 mM and 31.25 mM).

## Abbreviations

(PFTs) polyfunctional thiols; (*S.*) *Saccharomyces*; (γ-GluCys-) γ-glutamylcysteine; (G-) glutathionylated; (Cys-) cysteinylated; (CysGl-) cysteinylglycine; (°P) °Plato; (°C) °Celsius; (3SHol) 3-sulfanylhexanol; (3S4MPol) 3-sulfanyl-4-methylpentanol; (3SPol) 3-sulfanylpentanol; (γ-GT) γ-glutamyltranspeptidase; (CPY) carboxypeptidase; (NBT*p*Na) *N*-benzoyl-L-tyrosine-*p*-nitroanilide; (Tris-HCl) tris(hydroxymethyl)aminomethane; (DTT) dithiothreitol; (DMSO) dimethyl sulfoxide; (PMSF) phenylmethylsulphonyl fluoride.
