# Supplementary material for: Measuring the Carboxypeptidase and γ-Glutamyltranspeptidase Activities of Lager and Ale Yeasts to Assess Their Impact on the Release of Odorant Polyfunctional Thiols Through Fermentation
Source: Molecules. 2025 Jun 6;30(12):2491. doi: 10.3390/molecules30122491 (PMC12196205; doi:10.3390/molecules30122491)
Supplement: Supplementary file 1 [file molecules-30-02491-s001.zip › molecules-3617495-supplementary.pdf]

Supplementary Materials

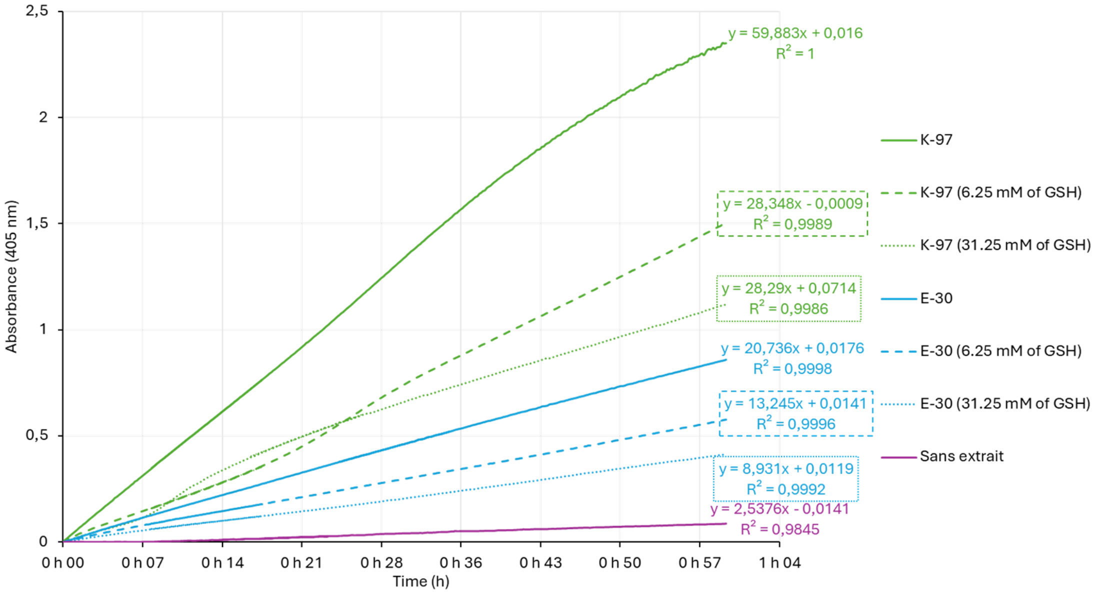

Figure S1. Enzymatic assays of γ-GT activity of K-97 and E-30 in presence of spiked glutathione (6.25 mM and 31.25 mM).
